# 

**Published:** 1910-07-30

**Authors:** 


### BOOKS RECEIVED.

Scientific Press, Ltd.

"Woman's Kingdom." By Mrs. W. Wallace.

"On the Evolution of Hospital Design." By K. D. Young, F.R.I.B.A.

"Medical Homes for Private Patients." By R. Pritchard Binnie.

S. M. H. K. & Co.

"Preservation of the Hair." By R. W. Leftwich, M.D.

Rebman, Ltd.

"Lectures on Cosmetic Treatment."

"Food and Hygiene." By Dr. Tibbles.

Macmillan and Co.

"Highways and Byways in Buckinghamshire." By C. Shorter, illustrated by F. L. Griggs.

Cassell and Co.

"Operative Surgery." By Treves and Hutchinson.

"A Manual of Chemistry." By A. P. Luff and H. C. R. Candy.

"Difficult Labour." By G. E. Herman.

"Movable Kidneys." By W. Billington, F.R.C.S.

### HOSPITAL FITTINGS.

### BED-PAN SINKS.

In our issue of July 2 we published an article on bed-pan sinks, and gave a descriptive account with illustrations of the various patterns in general use and of the firms who supply them. We have received a letter from Messrs. Twyford's Limited, in which they express surprise that no mention is made of their patterns. In our issue of April 9, p. 66, we did full justice to Messrs. Twyford's appliances and fixtures, and described them as all excellent models, giving prominence to their special appliances, including their mortuary table, portable bath, and lavatory appliances. The article on bed-pan sinks was meant to give an account of the original sinks, of which there are a number of variations from the original designs now in the market that we did not think it necessary to refer to. In addition to the sinks described and illustrated in our issue of July 2, the following firms make bed-pan sinks following the same lines as those illustrated: Messrs. Twyford's Limited (whose Cliffe Vale Pottery is very well known and very excellent), Messrs. Davies & Bennett, Messrs. Mellowes, and Messrs. Morrison, Ingram & Co.

### The Royal Visit to the London Hospital.

The King and Queen will visit the London Hospital this (Saturday) afternoon in order to inspect the wards and the institution generally.

Their Majesties will leave Marlborough House at a quarter to three o'clock in the afternoon, and will be accompanied by Prince George and Princess Mary, and by their suites. A travelling escort and mounted equerries will accompany the carriages, but troops will not line the route, which will be by way of the Mall, Holborn, Cheapside, and Whitechapel Road, returning by the South London route across London and Westminster Bridges. The King will be ceremoniously received at the City boundary at Holborn Bars, but so far as the London Hospital is concerned the visit will be a purely private one. Their Majesties are timed to arrive at the hospital gates at 3.10 o'clock, and various introductions will be made to them on their arrival. They will then proceed to some of the wards, and visit the $x$-Ray and the Finsen Light Departments. This is in accordance with the special desire of the King, who has asked to see certain new lamps in the Finsen Light Department, as well as the protective apparatus in the $x$-Ray Department, in which his Majesty is specially interested.

### COMING EVENTS OF THE WEEK.

[Communications for this column must be sent to 29 Southampton Street, Strand, before noon on Wednesday.]

Saturday, July 30.—The King visits the London Hospital, 3.10 p.m.

B.M.A. Reception at Bath.

B.M.A. Reception, Mayor Treloar's Cripples' Home.

Sunday, July 31.—Visiting day at the Hospitals.

Monday, August 1.—Sports in aid of German Hospital, Dalston.

French Neurological Congress opens. Brussels.

Tuesday, August 2.—International Congress on School Hygiene opens. Paris.

Thursday, August 4.—International Congress on Legal Medicine opens. Brussels.

League of Mercy. Buckinghamshire District. Lady Clayton "At Home," Harleyford, Marlow. 3.30 p.m.

The Last Word.—Thingummy and Whatshisname, two high priests of medicine, are without charity as regards each other, but the former is prudent in his comments, whereas the latter says what he thinks in unmeasured terms. "What is your opinion of your *confrère* Thingummy?" someone asked Whatshisname. "Thingummy? Why, he's an idiot, an ignoramus, a charlatan, and a disloyal fellow-worker!" "But you do him an injustice in calling him those names," replied the questioner, "for it was only the other day, on my asking him his opinion about you, that he unhesitatingly affirmed that he knew no one more intelligent, more learned, more loyal, and a better *confrère* than yourself." "Listen," answered Thingummy, in no way disconcerted. "Whathisname and I have made a compact together that, whenever an inquisitive individual asks one of us our opinion of the other, we shall always reply exactly the opposite to what we really think. That is what he did, and that is what I have just done also" (*L'Echo Médicale du Nord*).

### JOTTINGS.

His Majesty the King, while giving his patronage to the Seamen's Hospital Society (the "Dreadnought"), has been graciously pleased to intimate that he has increased the royal subscription to one hundred guineas per annum.

The Earl of Derby has laid the foundation stone of the new St. Paul's Eye Hospital, Liverpool. There will be a special department for the treatment of ophthalmia neonatorum to form a memorial to Dr. George Edward Walker, the founder of the original hospital of St. Paul's forty-one years ago.

The King has become patron of the following institutions: The Metropolitan Hospital Sunday Fund, Middlesex Hospital, University College Hospital, King Edward VII. Hospital, Windsor, the Royal Hospital for Diseases of the Chest, City Road, the Royal National Orthopædic Hospital, the Royal London Ophthalmic Hospital (Moorfields), the Royal Hospital for Incurables, Putney Heath, the Royal Ear Hospital, King's College Hospital, the Royal Sea Bathing Hospital, Margate, the Western Dispensary, Westminster, the Dorset County Hospital, Dorchester, the National Hospital for the Paralysed and Epileptic (Albany Memorial), Queen Square, Bloomsbury, the Metropolitan Convalescent Institution, and the Queen has become patron of the Middlesex Hospital, the Great Northern Central Hospital, and the Royal Hospital for Incurables, Putney Heath.

Mr. Vaughan Grey's matinée in aid of Prince Francis of Teck's appeal on behalf of the Middlesex Hospital, which was to be given at the Boudoir Theatre, Pembroke Gardens, on the 29th inst., has been unavoidably postponed until Thursday, November 10. We are asked to state that all tickets issued for Friday next will be available on the latter date.

At an influential meeting, convened by the Mayor (Councillor Fred Dyson), held at the Guildhall at Windsor last Tuesday to consider the question of a memorial to King Edward, Prince Christian, High Steward of the borough, presided, and was supported by Prince Alexander of Teck, Lord Burnham, Lord Haversham, Dr. Warre (Provost of Eton), Mr. P. J. Paravicini (Chairman of the hospital), and others. It was resolved to raise a fund by public subscriptions, and out of such fund to pay off the existing debt on the building and to erect a statue of his late Majesty in the grounds in front of the hospital.

The estate of Baron Shröder is another windfall for the estate duty section of the revenue. The estate duty amounts to about £312,000, which will be increased by legacy duties to about £480,000 under the present scale of the Finance Act. This compares with £315,000 under Mr. Asquith's Act of 1907 and with about £250,000 under the Act of 1894. Baron Schröder confirmed the gift by deed, dated July 26 last, to the Egham District Council of the site of an isolation hospital, and directed that if any duty be levied by reason of his death within twelve months (which occurred) his estate shall bear the duty.

### TO HOSPITAL SECRETARIES AND OTHERS.

We invite contributions from any of our readers, and shall be glad to pay, according to length, for items of news for the institutional section (including appointments, resignations, gifts, etc.) which we may publish. All payments for manuscripts used are made as early as possible after the beginning of each quarter.

### Gifts and Bequests.

Alderman Michael Huntbach, of Methven, Church Walks, Llandudno, bequeathed £50 to the Hanley Sick Nursing Association.

In response to Lord Denbigh's appeal for the Royal National Orthopædic Hospital £100 has been received from Mrs. Soper and £52 10s. from the Mercers' Company.

The Prince of Wales's General Hospital, Tottenham, has just received £250 from an anonymous source. This makes a total of £1,750 given to the hospital by the same person during the last three years.

The Right Rev. Dr. Edward King, Lincoln, Bishop of Lincoln since 1885, who died on March 8, aged eighty-one, left estate valued at £24,069 gross and £23,439 net. He bequeathed £500 to the Lincoln County Hospital.

Mr. Charles Crowther-Smith, of Anglesea House, Shirley, Southampton, left £50 each to the Royal South Hants and Southampton Hospital, the Shirley Children's Hospital and Dispensary for Women, and the Shirley Parochial Nurses' Fund.

Mr. Simon Leitner, of Warwick, has left £100 each to the Birmingham General Hospital, the Queen's Hospital, Birmingham, the Moseley Hall Convalescent Hospital for Children, the Evans Convalescent Home, Solihull; and £50 to the Brompton Cancer Hospital.

The Treasurer (Lord Sandhurst) gratefully acknowledges the receipt of the following further contributions towards the special fund of St. Bartholomew's Hospital: Mr. Anton Dunkels, £105; the Hon. Henry B. Portman, £100; Mr. Gardner Bazley, £100; Mr. J. Whateley Simmonds, £50; Sir Herbert B. Cohen, £50.

Mr. Thomas Hood Walker, of Ayrshire, and of London, a director of Messrs. John Walker and Sons (Limited), has left £4,000 to the Kilmarnock Fever Hospital and Infirmary, £1,000 each to the Ayr Infirmary and the Glenafton Sanatorium, and £3,000 for distribution by his trustees in their discretion among other charities in the county.

Prince Francis of Teck has received the following donations in response to his appeal on behalf of the funds of the Middlesex Hospital: Baron Bruno Schröder, Sir Alexander Henderson, and Mr. H. Osborne O'Hagan, £105 each; "E. J. M.," £100; and Messrs. Maple and Co., £105; Sir Everard Hambro, £500; Messrs. Speyer Brothers, £300; Mr. Pandeli Ralli, £100; Lady Bathurst, £100; "A Friend, per J. D. L.," £250.

Mr. Richard Peyton, of Edgbaston, Birmingham, formerly chemical manufacturer and associated with his father in the Birmingham and Midland (now the London City and Midland) Bank, has bequeathed, on the death of his wife, £3,000 to Birmingham General Hospital, £2,000 to Queen's Hospital, Birmingham, £3,000 to the Birmingham Midland Free Hospital for Sick Children (providing that the erection of the new hospital be begun within three years of the testator's death), £2,000 to the Birmingham and Midland Hospital for Women, £1,000 each to the Midland Counties Sanatorium, Blackwell, and the Birmingham General Dispensary, £500 each to the Birmingham and District Nursing Society, the Leamington Home for Incurables, the Ventnor Hospital for Consumption, the Birmingham and Midland Homœopathic Hospital, and the Birmingham and Midland Eye Hospital, £400 to the Birmingham Dental Hospital, £200 each to the Royal Orthopædic and Spinal Hospital, Birmingham, the Birmingham and Midland Skin and Urinary Hospital, the Birmingham and Midland Ear and Throat Hospital, and to the Lying-in Charity, Birmingham.

